# Novel Cyclovirus in Human Cerebrospinal Fluid, Malawi, 2010–2011

**DOI:** 10.3201/eid1909.130404

**Published:** 2013-09

**Authors:** Saskia L. Smits, Ed E Zijlstra, Jaap J. van Hellemond, Claudia M.E. Schapendonk, Rogier Bodewes, Anita C. Schürch, Bart L. Haagmans, Albert D.M.E. Osterhaus

**Affiliations:** ViroClinics BioSciences BV, Rotterdam, the Netherlands (S.L. Smits, A.D.M.E. Osterhaus);; Erasmus Medical Center, Rotterdam (S.L. Smits, J.J. van Hellemond;; C.M.E. Schapendonk, R. Bodewes, A.C. Schürch, B.L. Haagmans, A.D.M. E. Osterhaus);; College of Medicine, Blantyre, Malawi (E.E. Zijlstra)

**Keywords:** Paraplegia, virus, cyclovirus, random amplification, cerebrospinal fluid, serum, viruses, Malawi

## Abstract

To identify unknown human viruses, we analyzed serum and cerebrospinal fluid samples from patients with unexplained paraplegia from Malawi by using viral metagenomics. A novel cyclovirus species was identified and subsequently found in 15% and 10% of serum and cerebrospinal fluid samples, respectively. These data expand our knowledge of cyclovirus diversity and tropism.

The list of diseases caused by viral pathogens is ever changing and growing (*1*). Breakthroughs in the field of metagenomics had far-reaching effects on the identification of emerging viral pathogens and on the recognition that an increasing number of diseases that were once attributed to unknown causes are actually caused by infectious agents (*1*). Paraplegia is an impairment of motor or sensory functions of the lower extremities. Although it can be caused by spinal cord injury, nontraumatic paraplegia also should be considered, particularly in a tropical environment; tuberculosis and schistosomiasis may play a role, but in many cases, no firm diagnosis can be made (*2*). In this study, cerebrospinal fluid (CSF) and serum samples were obtained from 58 patients from Malawi who had paraplegia of unknown etiology and were studied for the presence of known or unknown viruses by using a metagenomics approach.

## The Study

During 2010–2011, we enrolled 58 adults who sought care at Queen Elizabeth Central Hospital in Blantyre, Malawi, for unexplained paraplegia in this study. All procedures were performed in compliance with relevant laws and institutional guidelines. The study was approved by the College of Medicine Research and Ethics Committee (P.99/00/92).

Serum and CSF samples obtained from 12 paraplegia patients were available for virus discovery studies by using random PCR amplification with next-generation sequencing with a 454 GS Junior Instrument (Roche, Indianapolis, IN, USA) (*3*,*4*). More than 234,000 trimmed reads were assembled by using de novo assembly in CLC Genomics Workbench 4.5.1 (CLC Bio, Aarhus, Denmark) and analyzed according to nucleotide and translated nucleotide BLAST searches (http://blast.ncbi.nlm.nih.gov/Blast.cgi). Classification of sequences based on the taxonomic origin of the best-hit sequence was performed by using MEGAN 4.40.4 (*5*), using E-value cutoffs of 0.001 and 10^−10^ for BLASTn and BLASTx searches, respectively.

Most of the sequences were of eukaryotic or bacterial origin or did not have hits to nucleotide or amino acid sequences in GenBank in agreement with previous viral metagenomic studies (*6*). Serum samples from all patients were positive for viruses from the family *Anelloviridae* in serum. Anellovirus infections are acquired during early childhood, when the virus establishes a chronic productive infection with long-lasting detectable viremia (*7*). Two of these patients were positive for anelloviruses in the CSF, which has been described (*7*). In 2 patients, hepatitis B virus sequences were detected. Hepatitis B virus infection occurs worldwide but is most prevalent in Southeast Asia and sub-Saharan Africa, with reported prevalence rates of 3%–26% (*8*). CSF from 2 other patients showed evidence of HIV infection in CSF samples (Table). None of these viral infections seem to explain the paraplegia.

**Table:** Viruses detected in serum and CSF samples from patients with paraplegia in a study of novel human cyclovirus, Malawi, 2010–2011*

Patient no.	Next-generation sequence			Cyclovirus	
	Serum	CSF		Serum	CSF
VS5700001	Anelloviridae				
VS5700002	Anelloviridae				+
VS5700003	Anelloviridae				
VS5700004	Anelloviridae, HBV				
VS5700005	Anelloviridae				
VS5700006	Anelloviridae	HIV-1			
VS5700007	Anelloviridae				
VS5700008	Anelloviridae, HBV	HIV-1, Anelloviridae			
VS5700009	Anelloviridae, Cyclovirus			+	+
VS5700010	Anelloviridae	Anelloviridae		+	
VS5700011	Anelloviridae				
VS5700012	Anelloviridae				
VS5700021	NA	NA		+	
VS5700022	NA	NA		NA	+
VS5700025	NA	NA		+	
VS5700029	NA	NA		+	
VS5700031	NA	NA		+	
VS5700040	NA	NA		+	NA
VS5700042	NA	NA		+	NA
VS5700044	NA	NA			+


*CSF, cerebrospinal fluid; +, positive; HBV, hepatitis B virus; NA, not available. Blank cells indicate negative results.

A cyclovirus genome was obtained by 454-sequencing from serum of patient VS5700009. Cycloviruses (family *Circoviridae*, genus *Cyclovirus*) have been detected in human and chimpanzee feces and tissues of farm animals, bats, and dragonflies (*9*–*12*). They are nonenveloped viruses with a single-stranded circular DNA genome of ≈2 kb (*13*). The genome contains 2 major inversely arranged open reading frames (ORFs) encoding the putative replication-associated protein (Rep) and capsid protein (Cap). A potential stem–loop structure with a conserved nonanucleotide motif located between the 5′-ends of these 2 ORFs is required to initiate the replication of the viral genome (*13*).

The genome organization of human cyclovirus VS5700009 (GenBank accession no. KC771281) resembles that of human cycloviruses TN18 and TN25 (*11*). The Rep ORF of human cyclovirus VS5700009 was interrupted by a 96-bp intron with a splice donor site (GT) and splice acceptor site (AG). The 3′ intergenic region between the Rep and Cap ORFs was only 7 bp long. The cyclovirus stem–loop structure with the conserved nonamer sequence (5′-TAATACTAT-3′) in the 5′ intergenic region was observed, as were 3 other potential ORFs (Figure, panel A, Appendix). These latter ORFs are not conserved among cycloviruses and show low partial homology to bacterial enzymes, NAD-dependent DNA ligase of *Psychrobacter* (ORF3), transketolase of *Sinorhizobium* (ORF4), and dTDP-D-glucose 4,6-dehydratase of *Actinomyces* (ORF5). The complete Rep protein of human cyclovirus VS5700009 and representative strains of cycloviruses were used for phylogenetic analysis (Figure, panel B, Appendix). Multiple alignments were created by using ClustalX (2.0.10) (*14*). Phylogenetic analyses were conducted with MEGA5 (*15*). The human cyclovirus VS5700009 Rep protein was most closely related to the Rep proteins of human cycloviruses TN18 and TN25 (≈75% aa identity) (*11*). Similar relations, but with much lower amino acid identities, were observed in Cap proteins (≈37% identity; data not shown). Cycloviruses belong to the same species when sharing >85% aa identity in the Rep region (*11*). Thus, human cyclovirus VS5700009 represents a new cyclovirus species. Most of the closest relatives of human cyclovirus VS5700009 (which include TN18 and TN25) detected in feces from children with nonpolio acute flaccid paralysis (*11*), a condition related to paraplegia, which may indicate that TN25/VS5700009-like viruses may be more pathogenic than other cycloviruses (Figure, panel B, Appendix).

**Figure:**
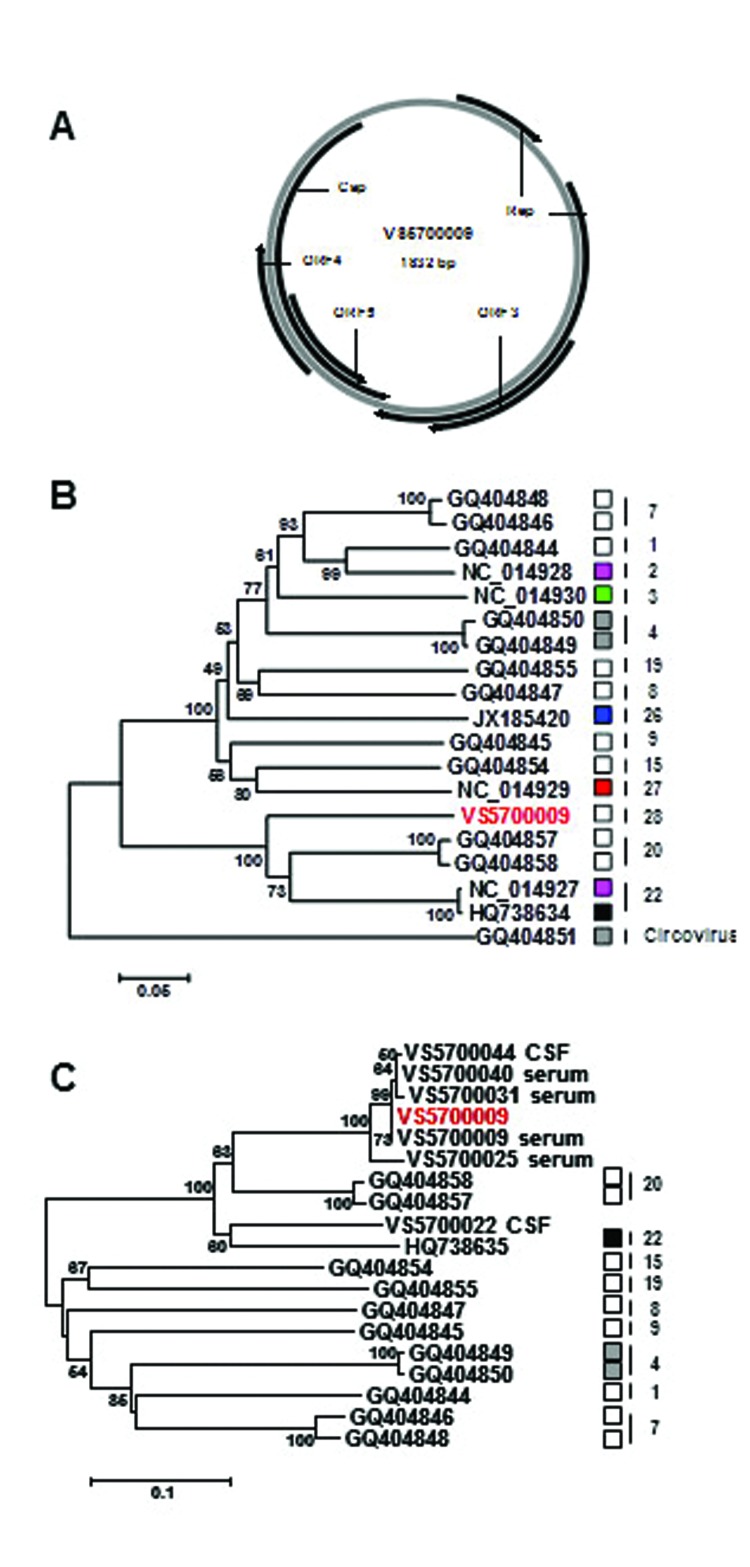
Cyclovirus genome organization and phylogenetic analysis of translated putative replication-associated protein (Rep) sequences. A) Size and predicted genome organization of human cyclovirus VS5700009, showing the 2 major open reading frames (ORFs) encoding Rep and the putative capsid protein (Cap, and other ORFs with a coding capacity >100 aa (ORFs 3–5). B) Phylogenetic tree of the translated Rep sequence of human cyclovirus VS5700009 and representative human and animal cycloviruses, generated by using MEGA5 with the neighbor-joining method with p-distance and 1,000 bootstrap replicates. A chimpanzee circovirus was used as an outgroup. Significant bootstrap values are shown. Cycloviruses in the same species are defined as having >85% aa identity in the Rep region and are labeled by vertical bars as described (*11*). The human (white), dragonfly (blue), bat (red), goat (pink), cattle (black), chicken (green), and chimpanzee (gray) cycloviruses are indicated, and VS5700009 is highlighted in red. Scale bar = 5% estimated phylogenetic divergence. C) Phylogenetic tree of the genomic area, corresponding to nt 392–733 in human cyclovirus VS5700009 and representative human and animal cycloviruses, generated by using MEGA5 with the neighbor-joining method with p-distance and 1,000 bootstrap replicates. Significant bootstrap values are shown. Cycloviruses isolated from the same species are labeled by vertical bars as described (*11*). Cycloviruses from humans (white), cattle (black), and chimpanzees (gray) are indicated, and VS5700009 is highlighted in red. Scale bar = 10% estimated phylogenetic divergence.

To determine the prevalence of human cyclovirus VS5700009 in the serum and CSF samples of the 58 patients, a VS5700009-specific PCR was performed. Total nucleic acid was extracted from an aliquot (≈100 μl) of serum and CSF samples by using the Magnapure LC total nucleic acid isolation kit and the MagNAPure LC isolation station (Roche). A genomic area, corresponding to nt 373–752 in human cyclovirus VS5700009, was amplified by nested PCR, with primers VS711 (5′-CGAGCGCACATTGAGAAAG-3′) and VS712 (5′-CCATCCCACCATTCTCCTC-3′) by using Amplitaq gold DNA polymerase (Roche). Negative water controls were taken along. Eight (15%) of 54 serum samples and 4 (10%) of 40 CSF samples from paraplegia patients were human cyclovirus positive (Table). For several amplicons, the cyclovirus nature was confirmed by Sanger sequencing, and the amplicons showed 75%–99% identity to human cyclovirus VS5700009 (Figure, panel C, Appendix). Only in patient VS5700009 were both CSF and serum samples positive for human cyclovirus.

## Conclusions

Our results indicate that cycloviruses are commonly found in serum and CSF of paraplegia patients from Malawi. Diverse cycloviruses have been discovered in human and chimpanzee fecal samples and in muscle tissue of farm animals, such as cows, sheep, goats, and chickens (*11*). Cycloviruses have been suggested to cause human enteric infections and were not derived from consumed food because the human and animal cycloviruses showed limited genetic overlap (*11*). Our data indicate that cycloviruses may cause systemic infections and are present in multiple organ compartments in humans. Whether cycloviruses play a role in development of paraplegia remains to be determined; this study lacks a control group of healthy persons, and the relatively high virus prevalence in persons with paraplegia may also reflect high overall prevalence in healthy persons. In addition, the apparent interleaved evolution of human and animal cycloviruses suggests the potential for frequent cross-species exposure and zoonotic transmission.

Our observations expand the knowledge of cycloviruses in humans and show how epidemiologic baseline information on virus host range and tropism in animals (*11*) may indicate the presence of similar viruses in different organ systems of humans. To clarify the epidemiology and pathogenicity of cycloviruses in humans, additional surveillance should be conducted, especially because the prevalence and diversity of human cycloviruses is relatively high (*11*; this study), cross-species transmission of cycloviruses seems plausible (*10*), and closely related cyclovirus species may be pathogenic.
